# Species Diversity Regulates Ecological Strategy Spectra of Forest Vegetation Across Different Climatic Zones

**DOI:** 10.3389/fpls.2022.807369

**Published:** 2022-03-02

**Authors:** Xin Han, Yue Xu, Jihong Huang, Runguo Zang

**Affiliations:** ^1^Key Laboratory of Forest Ecology and Environment of National Forestry and Grassland Administration, Institute of Forest Ecology, Environment and Nature Conservation, Chinese Academy of Forestry, Beijing, China; ^2^Co-innovation Center for Sustainable Forestry in Southern China, Nanjing Forestry University, Nanjing, China

**Keywords:** biotic factors, CSR theory, ecological strategy spectrum, species richness, stem abundance, vegetation type

## Abstract

Ecological strategy is the tactics employed by species in adapting to abiotic and biotic conditions. The ecological strategy spectrum is defined as the relative proportion of species in different ecological strategy types within a community. Determinants of ecological strategy spectrum of plant community explored by most previous studies are about abiotic factors. Yet, the roles of biotic factors in driving variations of ecological strategy spectra of forest communities across different geographic regions remains unknown. In this study, we established 200 0.04-ha forest dynamics plots (FDPs) and measured three-leaf functional traits of tree and shrub species in four forest vegetation types across four climatic zones. Based on Grime’s competitor, stress-tolerator, ruderal (CSR) triangular framework, and the StrateFy method, we categorized species into four ecological strategy groups (i.e., C-, S-, Int-, and R-groups) and related the ecological spectra of the forests to three species diversity indices [i.e., species richness, Shannon-Wiener index, and stem density (stem abundance)]. Linear regression, redundancy analysis, and variance partition analysis were utilized for assessing the roles of species diversity in regulating ecological strategy spectra of forest communities across different climatic zones. We found that the proportion of species in the C- and Int-groups increased, while the proportion of species in the S-group decreased, with the increase of three indices of species diversity. Among the three species diversity indices, stem abundance played the most important role in driving variations in ecological strategy spectra of forests across different climatic zones. Our finding highlights the necessity of accounting for biotic factors, especially stem abundance, in modeling or predicting the geographical distributions of plant species with varied ecological adaptation strategies to future environmental changes.

## Introduction

One of the challenges in ecological research is how to accurately predict future environmental changes on the abundances and spatial distributions of species, and the geographical patterns of biodiversity in the future (Cardinale et al., 2012; Urban et al., 2016). Changes in abiotic/biotic conditions or disturbance regimes can all impact population dynamics and community structure. Finding the likely driving factors of these changes is helpful in predicting the responses of species populations or biotic communities and in designing mitigation strategies to reduce the harmful effects of environmental change and human activities. Determinants on the distribution of species or plant communities explored by most of the previous studies are about abiotic factors (Harrison et al., 2020; Madrigal-González et al., 2020), yet evidence of how biotic factors shape the distributions of species or their assemblages at different spatial scales have received less attention (Araújo and Luoto, 2007). Ecological strategies, representing plants exhibiting similar adaptions to environmental conditions, can reflect tactics employed by species for growth and survival (Walker, 1992). An ecological strategy spectrum is actually the proportions of species with different ecological strategy types in a biotic assemblage. Similar to a life-form spectrum (Raunkiaer, 1934), it summarizes the enormous complexity of species with diverse ecological strategies into a few general recurrent groups. It can reflect combinations of species’ adaptation tactics coping with specific environmental conditions, which can provide an effective way for comparing how species composition of vegetation differs in their spatial distributions across different biogeographic regions and for detecting the key drivers of these differences. Consequently, exploring the impacts of biotic factors on ecological strategy spectra of forests can further enhance our understanding of the population adaptation performance of plant species and the resultant community features and improve our ability in predicting species responses to environmental changes.

A well-recognized species adaptation strategy scheme is Grime’s competitor, stress-tolerator, ruderal (CSR) theory (Grime, 1974, 1977, 2001; Grime and Pierce, 2012). Based on the trade-off among the competition, stress, and disturbance resulting from natural selection, three principal strategies characterized by the occurrence of combinations of different traits. Specifically, C-selected competitors are characterized with traits to aid resource preemption surviving in high-productivity habitat; S-selected stress-tolerators are characterized by traits in response to highly unproductive and abiotically variable habitat; and R-selected ruderals are characterized with traits in the face of frequent disturbances. This theory simultaneously explains both economics (a trade-off in resources investment in “acquisitive” to “conservative” growth) and size (organs and whole plants) as fundamental gradients of plant evolution (Diaz et al., 2016; Pierce and Cerabolini, 2018). Yet, due to the lack of a simple protocol for identifying the ecological strategies of species, the utilities of CSR theory have been debated for many years (Westoby, 1998; Craine, 2005). Since functional traits within and among plant species can provide a surrogate for some fundamental ecological processes (McGill et al., 2006), identifying species ecological strategies based on plant functional traits has received more and more attention (Pierce and Cerabolini, 2013; Diaz et al., 2016; Pierce et al., 2017). Based on functional traits, Grime’s CSR framework has been applied widely in ecological research (Caccianiga et al., 2006; Schmidtlein et al., 2012), e.g., examining species coexistence (Pierce et al., 2014) and correlation of species richness and productivity (Cerabolini et al., 2016). For promoting a wider range of applications and broader generalization of the CSR theory in biogeographically distinct contexts worldwide, Pierce et al. (2017) proposed a CSR ordination method (i.e., StrateFy). This method used only three-leaf functional traits, i.e., leaf area (LA), specific leaf area (SLA), and leaf dry matter content (LDMC), to classify plant species into 19 ecological strategy categories and ordinate them in CSR triangle. The protocol has been experimentally tested by manipulating resource availability and disturbance (Li and Shipley, 2017), and it has been used to investigate community assembly processes or vegetation responses to disturbance (Rosado and de Mattos, 2017; Han et al., 2021).

However, 19 ecological strategy categories may be too numerous for identifying the actual ecological responses of species to varying habitats and for clearly defining and explaining how each of the strategy categories responds to specific changes in environmental conditions across a large geographical scale. Consequently, we regrouped the species into only four ecological strategy groups, namely, C-strategy group (C-group), S-strategy group (S-group), R-strategy group (R-group), and intermediate-strategy group (Int-group). The regrouping allowed us to quantify how species were differentiated with environmental changes. To the best of our knowledge, previous studies have applied CSR theory and CSR strategy ordination method StrateFy to discern and compare the distribution of plant ecological strategies in responses to abiotic conditions, such as anthropogenic disturbance (Wang et al., 2018; Han et al., 2021), natural disturbances (Barba-Escoto et al., 2019; Matos et al., 2021), and climatic changes (Rosenfield et al., 2019). However, contrary to the effects of abiotic factors, studies on how biotic factors affect plant species strategies’ distributions at various spatial scales (e.g., landscapes, regions, and continents) have gained less attention in the background of predicting distribution patterns of vegetation in a changing environment.

Each species has its unique morphological, physiological, and phenological characteristics, i.e., having distinct functional traits, which make a species adapt to various environmental conditions. Communities are composed of species with different functional traits; thus, different species combinations in communities will affect their functions in ecosystems and the ecosystem services. Consequently, species-level analyses can provide insights into understandings at the community or ecosystem level (Symstad et al., 2003; Xu et al., 2019). In addition, species diversity is a key and common biotic driver of ecological processes and ecosystem functions (Tilman et al., 2014). Therefore, we selected three species diversity indices in this study, i.e., species richness, Shannon-Wiener index, and stem density (stem abundance), as possible biotic variables of driving variations in ecological strategy spectra of forest vegetation across different climatic zones. Species richness is a count of species within a biotic community, which is frequently used as the primary measure of biodiversity and is usually involved in other metrics of diversity (Burrascano et al., 2018). Abundance is an important measure of species diversity for evaluating species’ impact on local ecosystems (Ehrlén and Morris, 2015). It has also been incorporated into some indicators of biodiversity (Kenderes and Standovár, 2003). Shannon-Wiener index is a synthetic index, involving both richness and abundance to quantify distributions of individuals among different species in a community (Jabot and Chave, 2009; Bernhardt-Römermann et al., 2015). These biotic variables can reflect the impacts of plant interactions on plant community characteristics (Poorter et al., 2015). Hence, exploring the associations between ecological strategy spectrum and species diversity may provide insights into understandings on how biotic factors impact vegetation and their responses to changing environment.

Currently, one major obstacle in analyzing the associations between ecological strategy spectrum and species diversity is a lack of available data on vegetation investigation across different geographical regions. Here, we established 200 0.04-ha forest dynamics plots (FDPs) in four vegetation types of different climates and carried out a detailed investigation of woody plants (e.g., trees and shrubs) and measurement of their three-leaf functional traits. We calculated three species diversity indices (i.e., species richness, Shannon-Wiener index, and stem abundance) as potential biotic variables for driving variations of ecological strategy spectra for forests across different climatic zones. Based on CSR theory (Grime, 1974, 1977, 2001) and the CSR strategy ordination method StrateFy (Pierce et al., 2017), we identified species’ ecological strategies and ordinated them in the CSR triangle, thus obtaining the ecological strategy spectrum of each plot. Our objectives were to assess how species diversity impacted the variation of the ecological strategy spectra among different forest types by (1) assigning each species included in our data set into the CSR triangle to quantify the proportions of species in C-, S-, Int-, and R-groups in each forest type and then identify the ecological strategy spectra of forests across different climatic zones; (2) exploring the associations between the proportions of C-strategists, S-strategists, Int-strategists, and R-strategists with each species diversity index; and (3) assessing the relative importance of the three selected species diversity indices in regulating ecological strategy spectra of forests across different climatic zones. Rising diversity can lead to increasing competition among species (Grime, 2001; Aguilée et al., 2018). Since C-strategists and Int-strategists have more competitive ability than S-strategists characterized with the more tolerant ability for harsh conditions, we hypothesized that C-strategists and Int-strategists are positively associated while S-strategists are negatively associated with species diversity. Compared with the abundances of species and other biotic factors, the presence of species can more reflect variations of community compositions and environmental conditions across the earth (Kier et al., 2005; Mutke and Barthlott, 2005) and provide more to explain the origin and maintenance of species diversity (Currie et al., 2004; Weiser et al., 2007) across different climatic zones. Therefore, we hypothesize that species richness is the paramount biotic determinant for the variation of ecological strategy spectra of forests across different climatic zones.

## Materials and Methods

### Study Areas

This study was conducted in four provinces (i.e., Hainan, Hubei, Gansu, and Xinjiang), correspondingly distributed over the four climatic zones (i.e., tropical, subtropical, warm-temperate, and cold-temperate zone, respectively) across China. Four old-growth forest vegetation were investigated, namely, tropical forest located in the Bawangling forest region in Hainan Province, subtropical forest located in the Mulingzi and Xingdoushan forest regions in Hubei Province, warm-temperate forest in Xiaolongshan forest region in Gansu Province, cold-temperate coniferous forest in the Kannasi forest region in Xinjiang Province. The Mulingzi and Xingdoushan forest regions are two adjacent nature reserves in Hubei Province and both share similar climatic features. Additional detailed information of the study sites is shown in Supplementary Table 1.

### Data Collection

#### Vegetation Investigation

A total of 200 FDPs were established. At each study site, we established 50 0.04-ha (20 × 20 m) FDPs randomly. All the FDPs were established based on the CTFS standard.^1^ We ensured that the pairwise distance between each of the 0.04-ha plots centers was at least 200 m for avoiding spatial pseudo-replication among these plots. An autocorrelation analysis (Legendre and Legendre, 2012) for each forest type indicated no spatial autocorrelation existing among plots (Supplementary Figure 1). In each plot, we tagged and recorded all woody plants (e.g., trees and shrubs) individuals with diameter at breast height (DBH) ≥ 1 cm, including their identify, DBH, and spatial location. All plant specimens collected by our field investigations were deposited at the Herbarium of Chinese Academy of Forestry; the Herbarium of South China Botanical Garden, Chinese Academy of Sciences; and the Herbarium of Institute of Botany, the Chinese Academy of Sciences. All woody plant species were identified by our investigation team with the help of local botanists (including the Xiusen Yang in Hainan, Yongmei Yi in Hubei Province, and Anmin Li in Gansu). The plant species in Xinjiang were identified to species level by ourselves and local botanists at Xinjiang Academy of Forestry. All species involved in the present research were identified to species level. The species botanical nomenclature was standardized according to The Flora of China.^2^

#### Functional Traits

To identify each species’ ecological strategies, three-leaf functional traits, i.e., LA (mm^2^), SLA (mm^2^ mg^–1^), and LDMC (%), were measured for each species. The three traits were measured using standard methods (Pérez-Harguindeguy et al., 2013). For each species at each study site, we sampled at least 10 individuals. For species with fewer than 10 stems, we sampled all individuals. When the same species was present in two or more plots per forest type, we randomly sampled these species from different plots in each study site. Fully expanded and sun-exposed leaves were sampled in the field from each individual adult plant during the growing seasons (between June and September). To avoid unreliable measurements due to shrinkage of the leaves caused by plants dehydrating in the field, upon sampling, leaves-attached twigs were immediately put in sealed plastic bags and stored in prepared cool boxes. We then transported these samples to the laboratory and measured them within 24 h after collecting and rehydrating. All the species identified in our study were sampled, i.e., the number of species sampled is 243 in tropical forest, 171 in subtropical forest, 115 in warm-temperate forest, and 7 in cold-temperate forest.

In each individual sample, two to five expanded, healthy, pathogen- and pest-free leaves were chosen. Then fresh leaves were weighed for leaf fresh weight (LFW, mg) and scanned for LA. After weighing and scanning for fresh leaves, each leaf was dried in a hot oven at 80°C for 48 h and weighed to obtain leaf dry weight (LDW, mg). From the measurements of LFW and LDW and LA, we calculated SLA as the ratio of LA to LDW, and LDMC as the ratio of LDW to LFW. All procedures in terms of the sampling and measuring followed the scientific standard methods (Pérez-Harguindeguy et al., 2013). The data including the list of species used in this study and the mean values of functional traits can be available from https://datadryad.org/stash/share/pMTqaRJE9fwt0KZZwaW4NpnIEqqvxrNz67zGww_v_JM.

#### Diversity Indices

In this study, we utilized three diversity indices, including species richness, stem abundance, and Shannon-Wiener index as potential biotic factors. Based on the census data, we counted all species and all individuals in each FDP to obtain species richness and stem abundance for each FDP, respectively. Next, Shannon-Wiener index for each FDP was calculated with the number of individuals of each species present in each FDP (Liu et al., 2021).

### Data Analysis

#### Ecological Strategy Spectra of Forests

**FIGURE 1:**
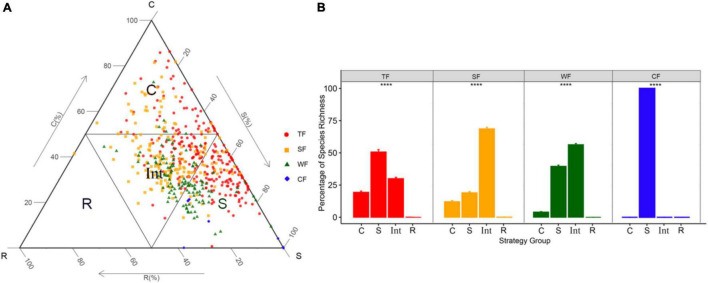
**(A)** The distribution of tree and shrub species (*n* = 536) and the delimitation of the four ecological strategy groups in the CSR triangle. **(B)** Bar plots showing species’ percentage of each ecological strategy group of ecological strategy spectrum of four forest types. ****P < 0.001 means significant difference among ecological strategy groups. Different symbols indicate species in different forest types: TF, tropical forest (red circles); SF, subtropical forest (yellow squares); WF, warm-temperate forest (green triangles); and CF, cold-temperate forest (blue diamonds). Ecological strategy group abbreviations are as follows: C, competitors; S, stress-tolerators; R, ruderals; Int, Int-strategists. Different colors indicate different forest types.

We utilized the CSR ordination method “StrateFy” (Pierce et al., 2017) using LA, LDMC, and SLA to identify species’ ecological strategy and ordinate them in the CSR triangle. To identify the ecological strategy spectra of forest communities, we connected the midpoints of each axis of the CSR triangle to divide the ternary plot into four parts. The four parts, respectively, corresponded to four resultant strategy groups: competitors ecological strategy group (C-group), stress-tolerators ecological strategy group (S-group), ruderals ecological strategy group (R-group), and intermediate-strategy ecological strategy group (Int-group; Figure 1). We then calculated the proportion of species occurring in each strategy group relative to the total number of species to further identify the ecological strategy spectrum of each forest type.

C-group included species with strong competitive abilities allowing them to dominate in habitats with relatively high productivity, S-group included species with higher stress tolerance maintaining individuals survival in variable and resource-poor environments, R-group included species with higher colonization *via* high fecundity, and Int-group is composed of species with characteristics intermediate between those of the competitor, the stress-tolerator, and the ruderal. We assumed that species in the intermediate-strategy group may tend to change into the other three ecological strategy groups when the habitats they survived experience different degrees of competition, stress, and disturbance. Specifically, when the habitat is in a strong biotic competition situation, the species in the intermediate-strategy group might change their adaptations toward the Competitive strategy. When the habitat is prevalent by stressful conditions, the species in the intermediate-strategy group might change their adaptations toward the Stress-tolerant strategy. And when the habitat is seriously disturbed, the species in the intermediate-strategy group might change their adaptations toward the Ruderal strategy.

We visualized the species’ position in the CSR triangle with “ggtern” function in “ggtern” package (Hamilton and Ferry, 2018) in R. Then, we performed generalized linear mixed model with the “glmer” function in the “lme4” package (Bates et al., 2015) for comparing species proportion in different ecological strategy groups.

#### Correlations Between Diversity Indices and Ecological Strategy Spectra

We used the “diversity” function in the “vegan” package to measure three diversity indices, namely, species richness, stem abundance, and Shannon-Winner index. Prior to data analysis, we used the variance inflation factor (VIF; “vif” function in “car” package; John and Sanford, 2019) in R to check the multicollinearity. A VIF > 10 suggested an excessive correlation between variables (Marini et al., 2011). The result of VIF revealed no multicollinearity between the biotic variables in our study (Supplementary Table 2).

To explore the association between biotic variables with ecological strategy group (i.e., C-, S-, Int-, and R-group), a generalized linear mixed model was used. We conducted models fitting on each ecological strategy group and each explanatory variable. Then, we calculated standardized regression coefficients (SRC) with the “apa.reg.table” function in the “apaTables” package (David, 2021) to compare the relative importance of the variables.

For assessing the relative impacts of the three biotic variables on the ecological strategy spectrum, we created two matrices, namely, the ecological strategy spectrum matrix (ecological strategy spectrum based on each plot) and biotic variables matrix (biotic variables based on each plot). Prior to the redundancy analysis (RDA; “rda” function in “vegan” package in R; Oksanen et al., 2015), the ecological strategy spectrum matrix was normalized by Hellinger transformation (Burrascano et al., 2018). The statistical significance of the effects of biotic variables on the ecological strategy spectrum was tested by Monte Carlo permutations (999 random permutations). Only significant (*P* < 0.05) variables were remained in the RDA model for further analysis. Based on the RDA result, we used the “varpart” function to partition variance for evaluating the relative influence of each biotic factor on the ecological strategy spectrum. All of the statistical analyses in this study were run in R software (v4.1.1).^3^

## Results

A total of 536 (243 in tropical forest, 171 in subtropical forest, 115 in warm-temperate forest, and 7 in cold-temperate forest) species were found across the four study sites. The ecological strategy of each species for each forest type was identified and plotted in the CSR triangle (see the different colored symbols in Figure 1A). These species occupied different positions in the CSR triangle, resulting in different proportions of species in each of the four ecological strategy groups, and thus forming different ecological strategy spectra for different forest types (Figure 1B). Specifically, in tropical forest, S-strategists accounted for the largest proportion, followed by Int-strategists and C-strategists. In subtropical forest and warm-temperate forest, Int-strategists were the most, followed by S-strategists and C-strategists. Cold-temperate forest involved only S-strategists. However, R-strategists only occurred in the subtropical forest.

**FIGURE 2:**
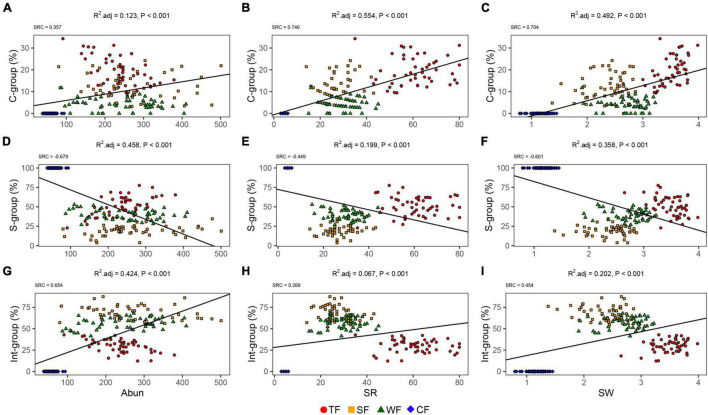
Relationship between ecological strategy group (i.e., C-, S-, and Int-groups) and diversity: species abundance (Abun; **A–C**), species richness (SR; **D–F**), Shannon-Wiener index (SW; **G–I**). Symbols in different colors and shapes indicate different forest types: TF, tropical forest (red circles); SF, subtropical forest (yellow squares); WF, warm-temperate forest (green triangles); and CF, cold-temperate forest (blue diamonds). Black lines indicate significant associations (*P* < 0.05).

The linear regression showed that correlations between the proportion of species in each ecological strategy group and each biotic factor differed (Figure 2). The proportions of species in C- and Int-groups were positively associated with species abundance, species richness, and Shannon-Wiener index. Conversely, the proportion of species in the S-group was negatively associated with all the abovementioned three species diversity indexes. For C-group, species richness (SRC = 0.746) and Shannon-Wiener index (SRC = 0.704) emerged as the most important variables (Figures 2A–C). Stem abundance (SRC = −0.679) was the main determinant for the proportion of species in the S-group, followed by the Shannon-Wiener index (SRC = −0.601, Figures 2D–F). Stem abundance (SRC = 0.654) and Shannon-Wiener index (SRC = 0.454) were the most important biotic variables explaining the proportion of species in Int-group (Figures 2G–I). Unlike the other three ecological strategy groups, the proportion of species in the R-group was not significantly affected by the biotic variables in this study (Supplementary Figure 2).

**FIGURE 3:**
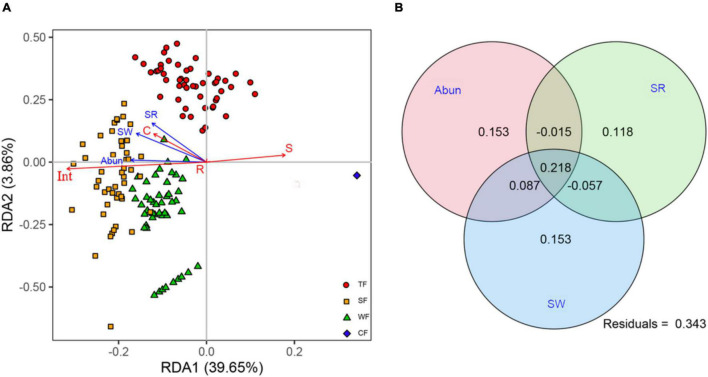
**(A)** Redundancy analysis (RDA) for ecological strategy spectra and biotic factors (blue arrows): stem abundance (Abun), species richness (SR), Shannon-Wiener index (SW). Symbols in different colors and shapes indicate species in different forest types: TF, tropical forest (red circles); SF, subtropical forest (yellow squares); WF, warm-temperate forest (green triangles); and CF, cold-temperate forest (blue diamonds). Ecological strategy group abbreviations (red arrows) are as follows: C, competitors; S, stress-tolerators; R, ruderals; and Int, Int-strategists. **(B)** Venn diagram showing variation partition analysis that partitions relative contributions of Abun, SR, and SW to ecological strategy spectra.

RDA revealed that our three biotic variables were clearly associated with ecological strategy spectra (adj-*R*^2^ = 65.71, *F* = 128.16, *P* < 0.001). Of the total variation in ecological strategy spectra, 43.51% was explained by the first two axes of RDA. The first RDA axis, explaining 39.65% variation in ecological strategy spectra, was negatively associated with stem abundance, species richness, and Shannon-Wiener index. The second RDA axis, explaining 3.86% of the variation in ecological strategy spectra, was positively associated with stem abundance, species richness, and Shannon-Wiener index (Figure 3A). The first two partial RDA axes as well as each of the variables independently had a significant loading (Supplementary Tables 3, 4), indicating that the biotic factors significantly affected variations in ecological strategy spectra of forests.

Variation partition analysis revealed that the variation in ecological strategy spectra explained by the three biotic variables amounted to 65.7%. Each of the variables independently accounted for a significant amount of variation in the ecological strategy spectra: stem abundance (44.3%), species richness (26.4%), and Shannon-Wiener index (40.1%; Figure 3B and Supplementary Table 5). These results suggested that stem abundance was the most important biotic variable in affecting the variation of ecological strategy spectra across different climatic zones.

## Discussion

We report the results from an extensive vegetation investigation across different climatic zones, analyzing ecological strategy spectra of tropical, subtropical, warm-temperate, and cold-temperate forests. This study revealed that species attributing to S-strategists presented the highest proportions in tropical and cold-temperate forests, while species belonging to Int-strategists occupied the highest proportion in subtropical and warm-temperate forests (Figure 1B). The proportions of species in C-, S-, and Int-groups were significantly associated with the three diversity indices (Figure 2), suggesting that species diversity significantly influenced ecological strategy spectra of forests across different climatic zones (Figure 3A). Furthermore, among the three biotic factors considered in this study, stem abundance was the most important biotic driver for variations of ecological strategy spectra of forests across different climatic zones (Figure 3B).

The proportion of species in the C-group increased as the species diversity increased (Figures 2A–C). We considered that favorable microclimatic conditions make more species coexist together, which could result in a more intensive competition among the coexisting species, leading to the higher proportions of C-strategists. Previous studies (Pretzsch, 2014; Jucker et al., 2015; Morin, 2015) have demonstrated that community with higher species diversity may characterize a more complex stand structure (Ehbrecht et al., 2017), which usually could buffer the heating and cooling effects of shortwave and longwave radiations (Häckel, 2008), creating a more favorable microclimate (Gebauer et al., 2012; Kunert et al., 2012; Forrester, 2015; Ehbrecht et al., 2017). Component with C-strategy was negatively associated with annual temperature range (Rosenfield et al., 2019) and temperature seasonality (Pierce et al., 2017). Benign or less stressful environments usually result in strong competition (the biotic filtering process) within and among species (Bertness and Callaway, 1994). These imply that favorable microclimate favors species with high competition ability survival and growth. In addition, species distributed in forest communities characterized with high species diversity usually possess higher competitive abilities (Aguilée et al., 2018). Ultimately, the proportion of C-strategists decreased from tropical forest through subtropical forest and warm-temperate forest to cold-temperate forest with decreasing species diversity. This finding indicated that certain species in forest communities with high species diversity would like to employ competitive ecological strategies to adapt to their living environment. In turn, the forest community with more C-strategists means higher species diversity, which may accompany a much higher conservation value.

The proportion of species in the Int-group was positively correlated with species diversity (Figures 2G–I). This may be due to the variable microhabitats resulting from higher species richness and stem abundance. Habitat-heterogeneity hypothesis has demonstrated that higher species diversity often leads to higher environmental heterogeneities within the forest communities (MacArthur and MacArthur, 1961). Higher environmental heterogeneities, in general, generate more variable microhabitats. For instance, light resource is a major limiting factor for the growth of trees. High diversity forests usually have more complex canopy structures, strongly impacting the distribution of light within forest communities (Kira et al., 1969). Each tree species usually has a characteristic crown architecture (Millet et al., 1998). In natural stands with higher species richness/stem abundance, trees present various growth forms, varying in size and crown architecture from understory through subcanopy to canopy or even overtop canopy species. This variation results in a multilayered foliage structure and enhances the structural complexity of the forest canopies, likely leading to the changes in light resources (Ishii et al., 2004) and thus the occurrence of various microhabitats occurring. Other than the effects of certain available resources due to species diversity, small-scale natural disturbance may be another important driver of environmental heterogeneities within forest community. Despite we have focused our sampling on old growth forests (i.e., in the late stage of succession) for minimizing the effects of disturbances on ecological strategy groups. However, natural disturbances are considered a crucial integral part of forest ecosystem dynamics and a major driver for species’ ecological strategies (Ehbrecht et al., 2021). For example, forests with higher species richness/stem abundance can provide more survival opportunities and more diverse food resources for more animal species (Southwood et al., 1979), especially for herbivores. In turn, different herbivores preferring different plant species or different aged stems (Lawton, 1983) cause a different degree of small-scale disturbance in a forest ecosystem. Meanwhile, high-diversity forest communities usually have more small-scale gap disturbances caused by death or breakage of large trees. Finally, uneven distribution of available light and small-scale disturbances may together create environmental heterogeneities and thus variable microhabitats in forest communities. Int-strategists characterized with intermediate between the competitor, the stress-tolerator, and the ruderal have higher adaptive capacity to the changing environmental conditions (Grime, 2001). Therefore, relatively higher stem abundance/species richness promote larger proportion of species in Int-group.

In contrast to the proportions of species in C- and Int-groups, the proportion of species in the S-group showed a negative correlation with species diversity (Figures 2D–F). This association between S-group and species diversity was believed to partly result from that S-strategists usually adapt to environments of high stress and low disturbance (Guo et al., 2019). In general, S-strategists have strong tolerance and resistance to stressful environments (Grime, 2001). Environmental stress (e.g., temperature) often restricts species richness (Wang et al., 2011), resulting in a higher proportion of S-strategists. In addition, S-strategists are characterized with low competitive ability for resources compared with C-strategists and their low tolerant ability to disturbances compared with Int-strategists (Pierce and Cerabolini, 2018). However, as we have discussed above, forest communities with higher species diversity theoretically favor the species with higher competitive ability or adaptive ability to variable microhabitats. Moreover, there are trade-offs among the C-, S-, and R-strategies according to the CSR theory, which suggests a compromise must be reached among the three in all species. For example, species with strong tolerance may show weak competitive ability (Bruelheide et al., 2018). Thus, we argued that trade-offs also existed among the C-, S-, Int-, and R-groups within forest communities for species coexistence. That is, the proportion of species in C- and Int-groups increased with species diversity may result in that in S-group decreased. Hence, the proportion of species in the S-group increased with the decrease of species diversity. For R-group and species diversity, as we expected, no significant correlations were found between them (Supplementary Figure 2). We assumed this may be due to the fact that the R-strategy is mostly adopted by herbaceous species, with only few woody species presenting the R-strategy in conditions with high disturbances (Rosenfield et al., 2019). However, we only focused on trees and shrubs and did not involve herbaceous species in this study.

Although all the three species diversity indices significantly affected the ecological strategy spectra of forest vegetation, their contributions were not equal in importance. Contrary to our hypothesis, variance partitioning revealed that stem abundance instead of species richness was the most important factor driving for variations of ecological strategy spectra among the three diversity indices (Figure 3B). This was in line with a previous study on the trait-demography relationships (Chalmandrier et al., 2021). Abundance was a much stronger indicator than the presence alone in species’ interactions in a community (Ehrlén and Morris, 2015). Furthermore, available space and resources for each individual as well as intra- and interspecific interactions to some extent can be reflected by stem abundance in communities (Fortunel et al., 2018). For example, high stem abundance was observed to have increased competition for light and space among individuals (Muscarella et al., 2018). In addition, the more individuals hypothesis (Gaston, 2000) also demonstrated that stem abundance can promote species richness (Madrigal-González et al., 2020). Shannon-Wiener index, which was second in importance of driving the variations in ecological strategy spectrum, is a complex combination of species abundance and stem richness (Jabot and Chave, 2009). These to some extent further confirmed the importance of stem abundance. Besides, the S-strategists and Int-strategists generally accounted for high proportions in the four forest types (Figure 1B) and were strongly affected by stem abundance (Figure 2). The above descriptions may together explain that stem abundance is paramount in promoting differentiations of ecological strategy spectra of forests across different climatic zones.

## Conclusion

Our study, for the first time, explored how species diversity impacted shifts in plant strategy spectra of vegetation based on CSR theory across four climatic zones. The findings clearly revealed that species diversity significantly drove the differentiation in ecological strategy spectra of forests across different climatic zones. For instance, species diversity increased C- and Int-strategists and decreased S-strategists. Evidence suggest that only considering the abiotic drivers is unlikely to accurately predict species distributions responses to future environmental changes (Morris et al., 2020). Changes in biotic drivers cannot be ignored as well (Wisz et al., 2013). Our findings further emphasize that biotic factors should be considered for better predicting how species distributions and vegetation variations respond to environmental change. However, contrary to our expectations, this study showed that stem abundance, not species richness, was the most important biotic driver for variations of forest ecological strategy spectrum. Although the study demonstrated that species diversity regulates the ecological strategy spectrum, some limitations in this study should be acknowledged. The major limitation lies in the fact that in this study, we only considered taxonomic diversity (e.g., species richness and abundance) as biotic factors, regardless of functional diversity. Therefore, more studies will be needed to better probe how biotic factors impact plant adaptive strategies in the future to improve our prediction capability for the dynamic of community response to global change. Despite this limitation, the research on CSR theory provided new insights into understanding the roles of species diversity in regulating the compositions of plant communities at regional scales under changing environmental context. This can also provide some guidelines for forest management and vegetation restoration under environmental change settings.

## Data Availability Statement

The raw data supporting the conclusions of this article will be made available by the authors, without undue reservation.

## Author Contributions

XH contributed to data analysis, manuscript drafting, and specimen identification. YX and JH performed the filed investigation and specimen identification. RZ designed the study. All authors participated in the writing.

## Conflict of Interest

The authors declare that the research was conducted in the absence of any commercial or financial relationships that could be construed as a potential conflict of interest.

## Publisher’s Note

All claims expressed in this article are solely those of the authors and do not necessarily represent those of their affiliated organizations, or those of the publisher, the editors and the reviewers. Any product that may be evaluated in this article, or claim that may be made by its manufacturer, is not guaranteed or endorsed by the publisher.

## Footnotes

1
http://www.ctfs.si.edu/

2
http://www.efloras.org

3
http://www.r-project.org/
