# Supplementary material for: Species Diversity Regulates Ecological Strategy Spectra of Forest Vegetation Across Different Climatic Zones
Source: Front Plant Sci. 2022 Mar 2;13:807369. doi: 10.3389/fpls.2022.807369 (PMC8924497; doi:10.3389/fpls.2022.807369)
Supplement: Supplementary file 1 [file Data_Sheet_1.docx]

**Supporting Information**

**Table. S1** The description of forest vegetation types across four climatic zones in China.

| Climatic zone | Forest vegetation type | Nature Reserve | Latitude (N) | Longitude (E) | MAT (°C) | MAP (mm) | No. of plot | No. of specie |
| --- | --- | --- | --- | --- | --- | --- | --- | --- |
| Tropical | Tropical rainforest | Bawangling | 18°52’-19°12’ | 108°53’-109°20’ | 23.6 | 1750 | 50 | 273 |
| Subtropical | Subtropical evergreen-deciduous broadleaved mixed forest | Mulingzi and Xingdoushan | 29°55’-30°10’ | 108°57’-110°17’ | 15.5 | 1733 | 50 | 171 |
| Warm-temperate | Warm-temperate coniferous-broadleaved mixed forest | Xiaolongshan | 33°30’-34°49’ | 104°22’-105°43’ | 10.9 | 800 | 50 | 115 |
| Cold-temperate | Cold-temperate coniferous forest | Kanasi | 48°35’-49°11’ | 86°54’-87°54’ | -0.2 | 1065 | 50 | 7 |

**Table S2.** The results of variance inflation factors (VIF) for three species diversity indices. Stem abundance (Abun), species richness (SR), Shannon Wiener index (SW).

| **Species Diversity Indices** | **VIF Value** |
| --- | --- |
| Abun | 1.63 |
| SR | 7.95 |
| SW | 8.34 |

**Table. S3** Summary of the permutation tests for significantly axis from redundancy analysis (RDA) for ecological strategy spectra in different forest vegetation types across climatic zones. Stem abundance (Abun), species richness (SR), Shannon Wiener index (SW).

| **RDA Axis** | **F value** | **P** |
| --- | --- | --- |
| RDA Axis 1 | 350.28 | 0.03 |
| RDA Axis 2 | 34.12 | 0.03 |

**Table. S4** Summary of the permutation tests for three species diversity indices from redundancy analysis (RDA) for ecological strategy spectra in different forest vegetation types across climatic zones. Stem abundance (Abun), species richness (SR), Shannon Wiener index (SW).

| **Species Diversity Indices** | **F value** | **P** |
| --- | --- | --- |
| Abun | 258.65 | 0.03 |
| SR | 36.75 | 0.03 |
| SW | 89.06 | 0.03 |

**Table. S5** Variation partitioning results of three species diversity indices based on redundancy analysis (RDA) for ecological strategy spectra in different forest vegetation types across climatic zones. Stem abundance (Abun), species richness (SR), Shannon Wiener index (SW).

| **Species Diversity Indices** | **adjusted R^2^** |
| --- | --- |
| Abun | 0.443 |
| SR | 0.264 |
| SW | 0.401 |

**Fig. S1:** Mantel Correlogram for spatial autocorrelation analysis. Mantel correlation between dissimilarity in ecological strategy spectra and distance among plots is shown for each distance class. Blank squares indicate that there were no significant spatial autocorrelations among the 50 plots within each of the same forest type. (TF, tropical rainforest; SF, subtropical forest; WF, warm-temperate forest; CF, cold-temperate forest).


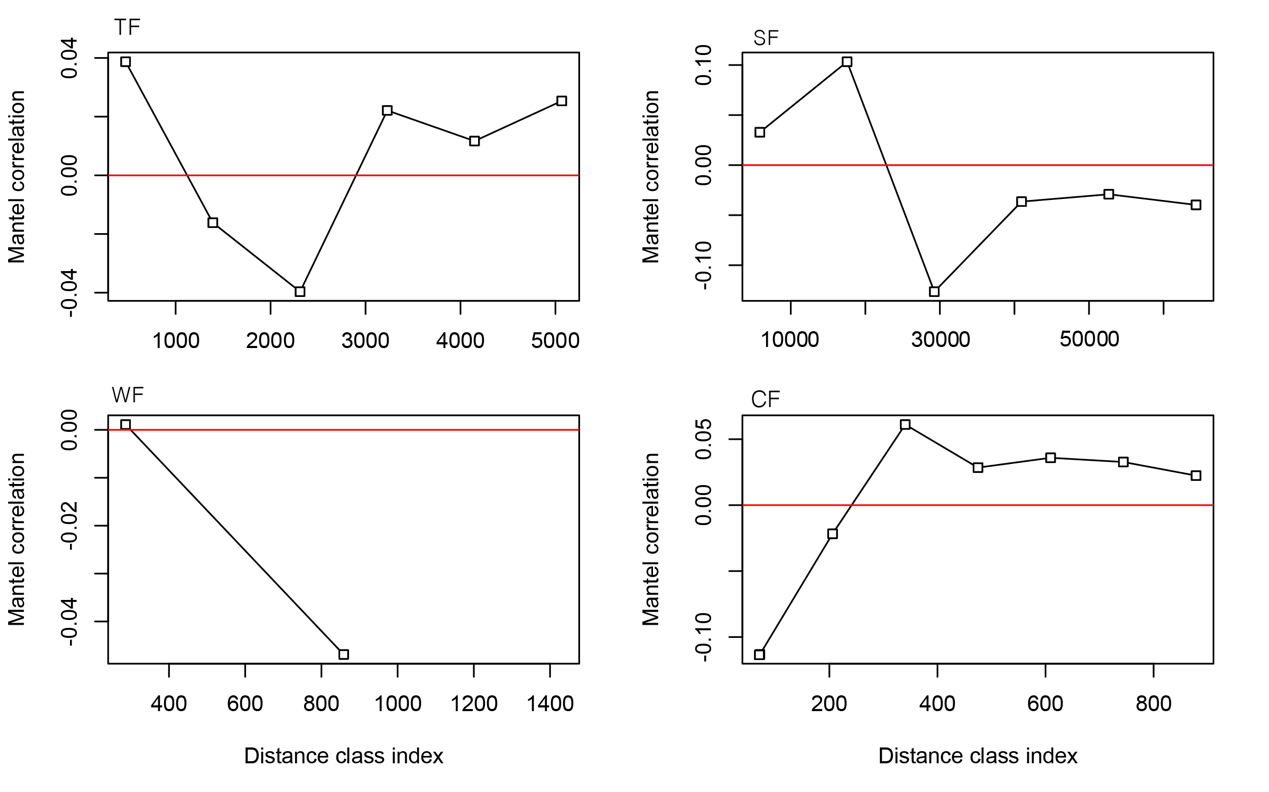


**Fig. S2:** Relationship between R-strategy group (R-group) and diversity: species abundance (Abun; A, species richness (SR; B), Shannon Wiener index (SW;C). Symbols in different colors and shapes indicate different forest types: TF, tropical forest (red circles); SF, subtropical forest (yellow squares); WF, warm-temperate forest (green triangles); CF, cold-temperate forest (blue diamonds). No lines indicate nonsignificant associations (p > 0.05).


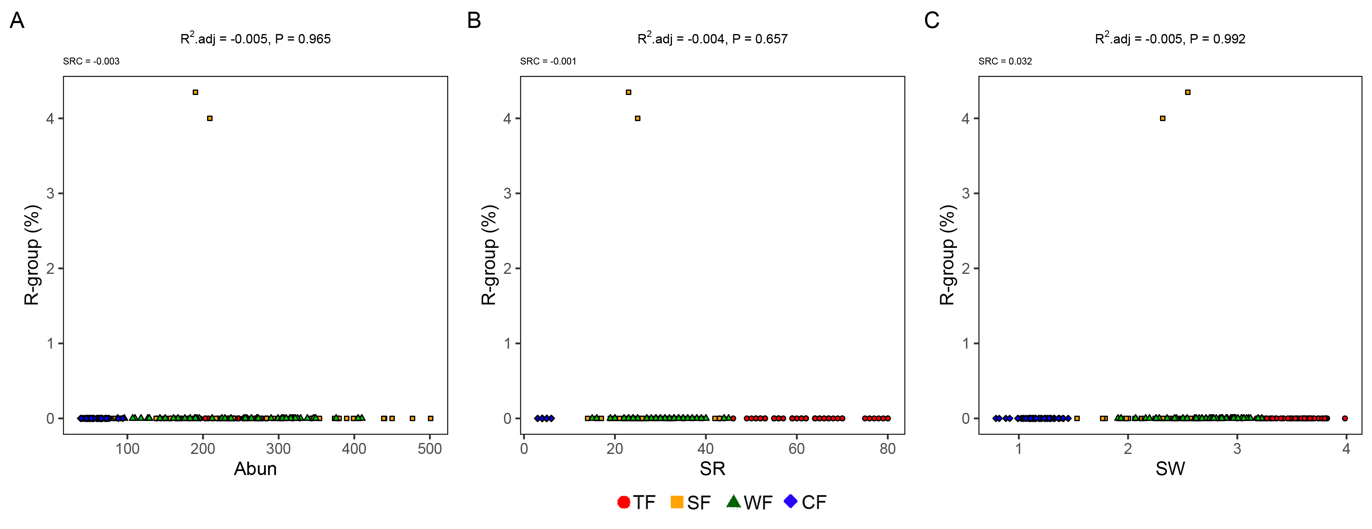


Fig. S3. A map with the locations of forest types across four climatic zones of China. CF (blue diamonds), cold-temperate coniferous forest in Kanasi of Xinjiang; WF (green triangles), warm-temperate coniferous and broadleaved mixed forest in Xiaolongshan of Gansu in; SF (yellow squares), subtropical evergreen-deciduous mixed forest in Mulingzi and Xingdoushan of Hubei; TF (red circles), tropical montane rain forest in Bawangling of Hainan.


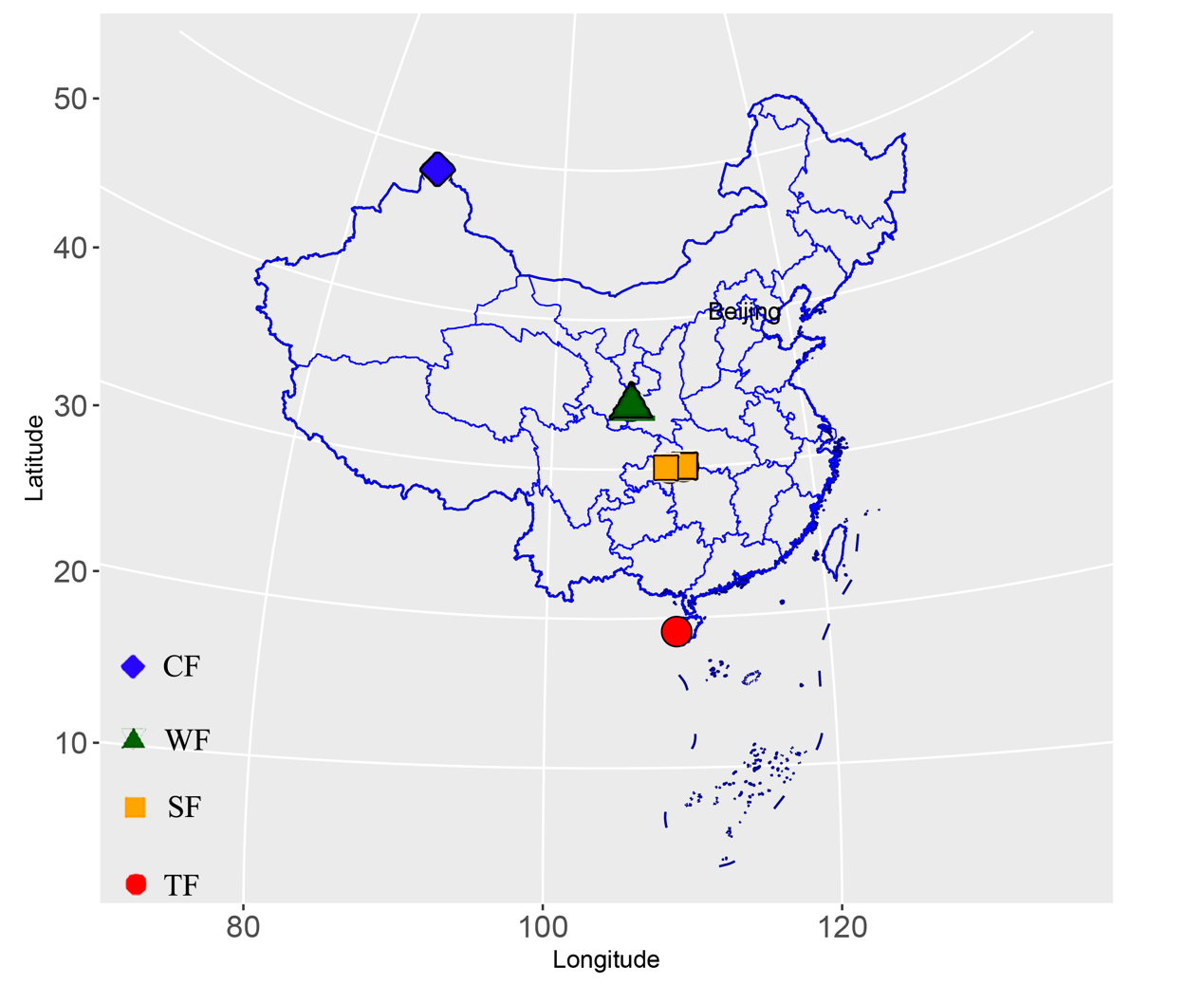


**Appendix. S1: Details of codes used in the study**

**1) The codes for Ternary Plot**

Data-data.frame, i.e., species and the mean values of C(%), S(%), R(%).

library (ggtern)

dev.new (

title = "CSR Triangle",

width = 4,

height = 4,

noRStudioGD = TRUE

)

Data$group<-factor(Data$group,levels=c("TF","SF","WF","CF"))

TER <- ggtern (data= Data, aes (x=R,y=C,z=S)) +

geom_tri_tern (bins=2, show.legend = F) +

geom_mask ()+

geom_point(aes(shape=group,color=group),size = 1.5,show.legend = F,alpha=1)+

scale_shape_manual(values = c(21,22,24,23)) +

scale_color_manual(values = c('red', 'orange', 'darkgreen',"blue")) +

theme_bw (base_size =10, base_family = "")+

theme (tern.panel.grid.major.T=element_line (colour = "white",size=0.7),

tern.panel.grid.major.L=element_line (colour = "white",size=0.7),

tern.panel.grid.major.R=element_line (colour = "white",size=0.7),

tern.panel.grid.minor.show = F)+

theme_ticklength (unit (5.0, 'mm'))+

labs ( x = "R",

xarrow = "R(%)",

y = "C",

yarrow = "C(%)",

z = "S",

zarrow = "S(%)",

title="Forest Type")+

theme_showarrows () +

theme_clockwise ()

TER

**2) The codes for Barplot**

library(lme4)

library(reshape2)

library(ggpubr)

Data-data.frame, i.e., “Spectra”, the data frame of the ecological strategy spectra matrix

model_spectra <- glmer (value~ Strategy + (1|type), data= Data)

model_spectra

summary(model_spectra)

bb1 <- melt (Data)

dev.new (

title = "Strategies",

width = 8,

height = 4,

noRStudioGD = TRUE

)

bb1$Group<-factor (bb1$Group, levels=c("C","S","Int","R")) # Arrange the order of Strategy Groups

Barplot <- ggbarplot (bb1, x = "variable", y = "value",

order= Group, add = "mean_se",

position = position_dodge (0.8),

ylim=c (0,100),

xlab = "Strategy Groups",

ylab = "Percentage of Species Richness",

title = " Strategy Group")

Barplot <- Barplot + stat_compare_means (aes (group = Group),

paired = TRUE,

label = "p.signif",

label.y =100)

Barplot

**3) The codes for RDA**

library(vegan)

Data.frame 1: Spectra - [ecological strategy spectra matrix] --Response variables

Data.frame 2: div- [diversity variables matrix] --Explained variable

Spectra <- decostand (Spectra, method = 'hellinger') # normalized by the Hellinger transformation

perform

rda_tb <- rda (Spectra ~., div, add=T)

#Forward selection procedures to select the main environmental variables.

rda_tb_forward_r <- ordiR2step (rda (Spectra ~1, div, scale = FALSE),

scope = formula(rda_tb), R2scope = rda_adj,

direction = 'forward', permutations = 999)
#Permutation test for overall model test

anova (rda_tb_forward_r)

summary (rda_tb_forward_r, scaling = 2)

#Read the information for plot the ordination plot

rda_tb_forward_r.scaling2 <- summary(rda_tb_forward_r, scaling = 2)

rda_tb_forward_r.site <- data.frame(rda_tb_forward_r.scaling1$sites)[1:2]

rda_tb_forward_r.div <- data.frame(rda_tb_forward_r.scaling1$biplot)[1:2]

#Add the group

group <- read.delim('group.txt', sep = '\t', stringsAsFactors = FALSE, check.names = FALSE)

rda_tb_forward_r.site$sample <- rownames(rda_tb_forward_r.site)

rda_tb_forward_r.site <- merge(rda_tb_forward_r.site, group, by = 'sample')

rda_tb_forward_r.div$sample <- rownames(rda_tb_forward_r.div)

#Perform ggplot2 to plot the ordination plot

library(ggplot2)

dev.new(

title = "Strategies ",

width = 4,

height = 4,

noRStudioGD = TRUE

)

rda_tb_forward_r.site$group<-factor(rda_tb_forward_r.site$group,levels=c("TF","SF","WF","CF"))

p <- ggplot(rda_tb_forward_r.site, aes(RDA1, RDA2)) +

geom_point(aes(fill = group,shape = group)) +

scale_shape_manual(values = c(21,22,24,23)) +

scale_fill_manual(values = c('red', 'orange', 'green3',"blue") ) +

theme(panel.grid = element_blank(), panel.background = element_rect(color = 'black', fill = 'transparent'), plot.title = element_text(hjust = 0.5), legend.key = element_rect(fill = 'transparent'))+

labs(x = 'RDA1 (39.65%)', y = 'RDA2 (3.86%)', title = 'RDA', color = '') +

geom_vline(xintercept = 0, color = 'gray', size = 0.5) +

geom_hline(yintercept = 0, color = 'gray', size = 0.5) +

geom_segment(data = rda_tb_forward_r.div, aes(x = 0, y = 0, xend = RDA1*0.2,yend = RDA2*0.2), arrow = arrow(length = unit(0.1, 'cm')), size = 0.3, color = 'blue') +

geom_text(data = rda_tb_forward_r.div, aes(RDA1 *0.2* 1.2, RDA2*0.2 * 1.2, label = sample), color = 'blue', size = 3)+

geom_segment(data = rda_tb_forward_r.sp, aes(x = 0, y = 0, xend = RDA1 *0.2,yend = RDA2 *0.2), arrow = arrow(length = unit(0.1, 'cm')), size = 0.3, color = 'red') +

geom_text(data = rda_tb_forward_r.sp, aes(RDA1 *0.2* 1.2, RDA2 *0.2* 1.2, label = sample), color = 'red', size = 3)

p

**Reference website**: https://mp.weixin.qq.com/s/pR_ZuGIjXzqg22Drdstc4w

**4) The codes for variance decomposition analysis**

div <-data.frame(scale(div))

part.all<-varpart(Spectra, div[c("Abun")],div[c("SR")], div[c("SW")])]

dev.new(

title = "Strategy Spectrum",

width =6,

height =6,

noRStudioGD = TRUE

)

showvarparts(2, bg=2:4)

plot (part.all,bg=2:5,cutoff=-1,digits=2)

**5) The codes for line regression**

Data-data.frame, i.e., div.spectra--the data frame of the ecological strategy spectra matrix combining with species diversity matrix.

library(lme4)

library(nlme)

library(ggplot2)

model <- glmer (srtategy~div + (1|plot), data = Data)

model

summary(model)

Data.scale<-data.frame(scale(Data))

model.scale<-lm(srtategy~div, data = Data.scale)

model.scale

summary(model.scale)

dev.new(

title = "Forest Plot",

width = 4,

height = 4,

noRStudioGD = TRUE

)

Data$group<-factor(Data$group,levels=c("TF","SF","WF","CF"))

p.model <-ggplot(Data, aes(div, srtategy))+

geom_point(aes(fill = group,shape = group),size = 1, alpha =1)+

scale_shape_manual(values = c(21,22,24,23)) +

scale_fill_manual(values = c('red', 'orange', 'green3',"blue") ) +

theme(panel.grid = element_blank(),

panel.background = element_rect(color = 'black',

fill = 'transparent'),

plot.title = element_text(size = rel(0.7),hjust = 0.5),

plot.subtitle = element_text(size = rel(0.5)),

legend.key = element_rect(fill = 'transparent'),

legend.position = 'none') +

geom_abline(aes(intercept = model$coefficients, slope = model$coefficients))+

labs(y = 'strategy (%)',

title = expression(R^{2}*'.adj = Adjusted R-squared, P < p-value '),

subtitle="SRC = model.scale$coefficients"

tag = "A")

p.model
